# Plasma tau and neurofilament light chain as biomarkers of Alzheimer's disease and their relation to cognitive functions

**DOI:** 10.25122/jml-2022-0251

**Published:** 2023-02

**Authors:** Sadiruldeen Sami Abed, Farqad Bader Hamdan, Mahir Mohammed Hussein, Qasim Sharhan Al-Mayah

**Affiliations:** 1 Department of Pharmacy, Osol Aldeen University College, Baghdad, Iraq; 2 Department of Physiology, College of Medicine, Al-Nahrain University, Baghdad, Iraq; 3 Ibn-Rushed Psychiatric Teaching Hospital, Baghdad, Iraq; 4 Medical Research Unit, College of Medicine, Al-Nahrain University, Baghdad, Iraq

**Keywords:** Alzheimer’s disease, neurofilament light chain, total tau protein, ADAS-cog

## Abstract

Alzheimer's disease (AD) dementia is the most frequent cause of neurodegenerative dementia. The cognitive and behavioral symptoms associated with this disorder often have overlapping characteristics, potentially resulting in delayed diagnosis or misdiagnosis. This study aimed to assess the level of peripheral blood neurofilament light chain (NfL) and total tau (t-tau) protein in AD patients and investigate their relationship with cognitive impairment. The study included 80 participants of both sexes between the ages of 60 to 85 years. The participants were divided into two groups, consisting of 40 individuals in the control group (mean age 75±6.6 years) who had no cognitive or functional impairments and 40 AD patients (mean age 74.98±5.03 years). This study utilized the DSM-5 diagnostic criteria for major or mild neurocognitive disorder attributed to Alzheimer's disease (AD). The clinical and biochemical features of all participants were documented, and the Alzheimer's disease Assessment Scale cognitive subscale (ADAS-cog) scores were evaluated. Sandwich ELISA was employed to determine serum NfL and t-tau protein levels. The median serum NfL and t-tau protein levels in AD patients were significantly higher than those of the controls (47.84 pg/ml versus 17.66 pg/ml and 12.05 pg/ml versus 11.13 pg/ml, respectively). Age was positively correlated with NfL, t-tau levels, and ADAS-cog. Although elevated NfL and t-tau protein levels may play a role in disease progression, their diagnostic value for AD was limited.

## INTRODUCTION

Plasma neurofilament light (NFL) chain and total Tau (t-tau) proteins have emerged as two potential biomarkers for the detection of Alzheimer's disease (AD) dementia. T-tau levels increase in pathology-confirmed AD and are recognized as a neurodegeneration biomarker in contemporary AD dementia research frameworks [1]. On the other hand, plasma NfL increases upon neuronal injury and correlates with clinical progression and survival in AD syndromes [2,3].

Research has indicated that blood t-tau has limited diagnostic potential for AD [4], whereas NfL in blood displays a significant increase in AD dementia [2]. Furthermore, NfL tracks neurofibrillary tangle load and cognitive decline [5,6]. Ultrasensitive measurement technologies have shown that both plasma t-tau and NFL have good/fair diagnostic accuracy in differentiating cognitively healthy individuals from those with AD [7-9]. Additionally, both biomarkers are associated with a decline in cognitive performance [2,6], cerebral atrophy [2,6,10], and hypometabolism [2,6] during the prodromal and dementia phases of AD.

Limited data is available regarding the impact of critical biological factors, such as age and sex, on plasma NfL and t-tau levels [11]. The objectives of this study were to (1) assess the level of peripheral blood NfL and t-tau protein in patients with AD dementia, (2) investigate their relationship with cognitive impairment (CI), and (3) compare the diagnostic value of plasma NfL and t-tau for AD dementia.

## MATERIAL AND METHODS

### Study population

An observational case-control study was carried out at the Ibn-Rushed Psychiatric Teaching Hospital, Baghdad, including 80 Iraqi participants of both sexes aged between 60 to 85 years. The participants were divided into two groups: 40 patients with clinically diagnosed AD (mean age 74.98±5.03 years) referred by a psychiatrist blinded to the biomarker results with no other psychiatric disorders, and a control group consisting of individuals without any psychiatric disorders.

The DSM-5 diagnostic criteria for major or mild neurocognitive disorders attributed to AD dementia was used. Patients had a positive family history of AD dementia with clear evidence of a decline in memory and learning based on detailed history and neurocognitive assessment by the Mini-Mental State Examination. In addition, the patients were well-established cases and had been visiting the hospital regularly. Furthermore, AD dementia patients had no evidence of mixed etiology (i.e., absence of other neurodegenerative or cerebrovascular disease or another neurological, mental, or systemic disease or condition likely contributing to cognitive decline).

The other group represented the healthy control group, comprising 40 age- and sex-matched subjects (mean age 75±6.6 years). They were functionally intact with no cognitive impairment on the Mini-Mental State Examination. Individuals with chronic diseases, such as autoimmune and neurodegenerative disorders, were excluded from the study.

### AD Assessment Scale-Cognitive Subscale Test

All the participants in the study were subjected to the same study protocol and assessed by a psychiatrist with experience in administering the Alzheimer's Disease Assessment Scale - Cognitive Subscale (ADAS-cog) to evaluate cognitive function. The version used in this study was the basic ADAS-Cog with 11 items. This test takes about 30 minutes and is scored from 0 to 70: higher scores indicate greater cognitive impairment (CI). The main areas of the cognitive domains evaluated are memory (50%), language (28%), praxis (14%), and command understanding (8%). A high score indicates poor performance [12].

### Immunological assay

Three milliliters of venous blood samples were collected in gel tubes in the morning, after 12-14 hours of fasting, from all participants by a certified laboratory technician. The samples were centrifuged at 3000 rpm for 10 minutes, and the resulting sera were frozen at -20°C until further processing. For the quantitative measurement of NfL and the t-tau protein, an enzyme-linked immunosorbent assay (ELISA) was used (Demeditec Diagnostics/Germany). The microtiter plate reader was set to read at a wavelength of 450 nm.

### Statistical analysis

The statistical analyses were conducted utilizing SPSS software version 25.0 (SPSS, Chicago). Continuous data were evaluated for normality using the Shapiro-Wilk test. Data that exhibited normal distribution were presented as the mean and standard deviation and were analyzed using an independent t-test. For data that were not normally distributed, a natural log transformation was applied and then converted to Z-scores, after which an independent t-test was performed. Categorical variables were presented as numbers and percentages and analyzed with the Chi-square test. To assess the discriminative ability of NfL and t-tau proteins in distinguishing AD from controls, the receiver operating characteristic curve (ROC) was utilized. A combination of both biomarkers was used in the logistic regression model. The cut-off value with the highest sensitivity and specificity was calculated according to Youden Index [13]. The correlation of NfL, t-tau and ADSA-cog with age and BMI was explored by Pearson’s correlation test after transforming the data into Z-score. A probability equal to or less than 0.05 was considered significant.

## RESULTS

### Demographic characteristics of the study population

This study included 40 patients with AD and 40 healthy controls. Patients had a mean age of 74.98±5.03 years, which was not significantly different from the controls (75±6.6 years). Similarly, the sex distributions of the two groups were comparable, although females were more numerous among patients. The mean BMI of patients with AD was 21.12±2.65 kg/m^2^, significantly lower than the mean BMI of controls (26.14±3.37 kg/m^2^) with a highly significant difference. In addition, patients with AD exhibited a significantly higher ADAS-cog score than controls (48.73±7.8 *vs*. 4.95±1.61), with a highly significant difference (Table 1).

**Table 1:** Demographic data of the study population.

Variable	Study groups		P-value
	AD dementia patients	Controls	
**Age at inclusion, years**			
Mean±SD	74.98±5.03	75.03±6.59	0.097
Range	67–85	62–88	
**Age at onset, years**			
Mean±SD	69.55±4.71		
Range	62–79		
**Sex**			
Males	17 (42.5%)	21 (52.5%)	0.30
Females	23 (57.5%)	19(57.5%)	
**BMI kg/m^2^**			
Mean±SD	21.12±2.65	26.14±3.37	<0.001
Range	15.97–26.38	19.22–32.03	
**ADAS-cog**			
Mean±SD	48.73±7.8	4.95±1.61	<0.001
Range	38–68	2.0–8.0	

AD – Alzheimer’s disease.

### NfL and t-tau proteins

**Figure 1:**
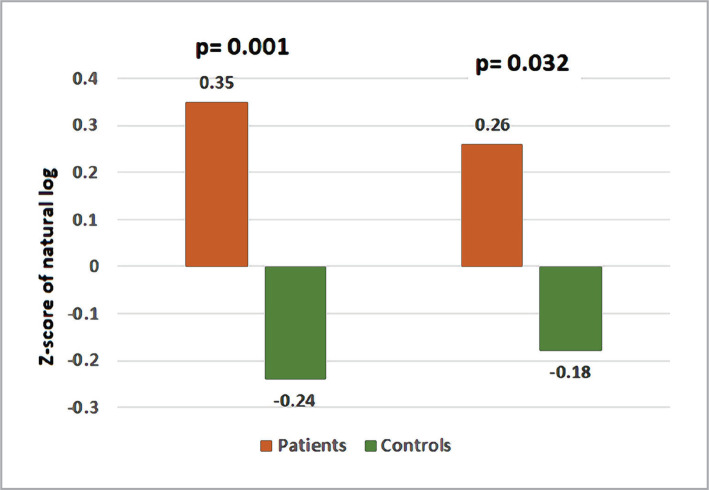
Z-score of the natural log of NfL and t-tau proteins in AD patients and controls.

The mean Z-score of the natural log of serum level of t-tau protein in patients with AD dementia was 0.35±0.11, significantly higher than that of controls (-0.24±0.15) with a highly significant difference. Similarly, patients with AD dementia showed a significantly higher mean Z-score of the natural log of NfL (0.26±0.14) than controls (-0.18±0.09) with a significant difference (Figure 1).

### Diagnostic value of NfL and t-tau

**Figure 2:**
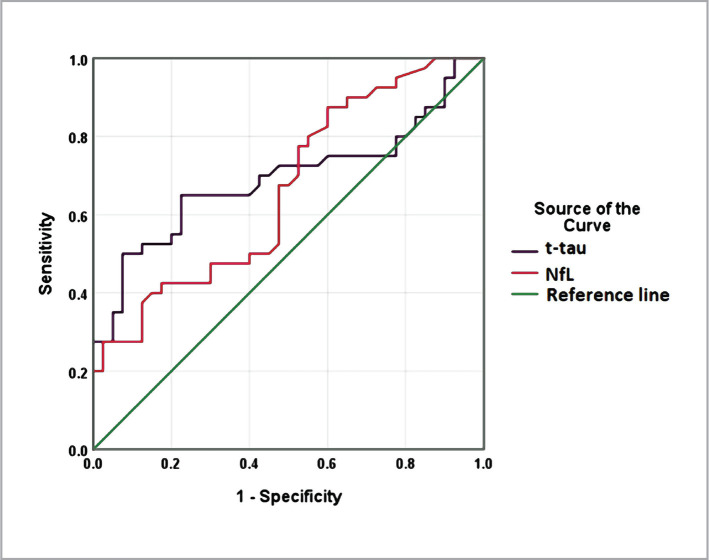
ROC curve for NfL and t-tau and neurofilament in the context of discrimination between patients with AD and controls.

The AUC for t-tau protein was 0.693 (95% CI: 0.572-0.813, p=0.003). At a cut-off value of t-tau= 20.61 pg/ml, the sensitivity and specificity of the test were 0.68 and 0.57, respectively. The AUC for NfL was 0.664 (95% CI:0.546-0.783, p=0.011). At a cut-off value of NfL= 11.21 pg/ml, the sensitivity and specificity of the test were 0.68 and 0.52, respectively (Figure 2).

**Figure 3:**
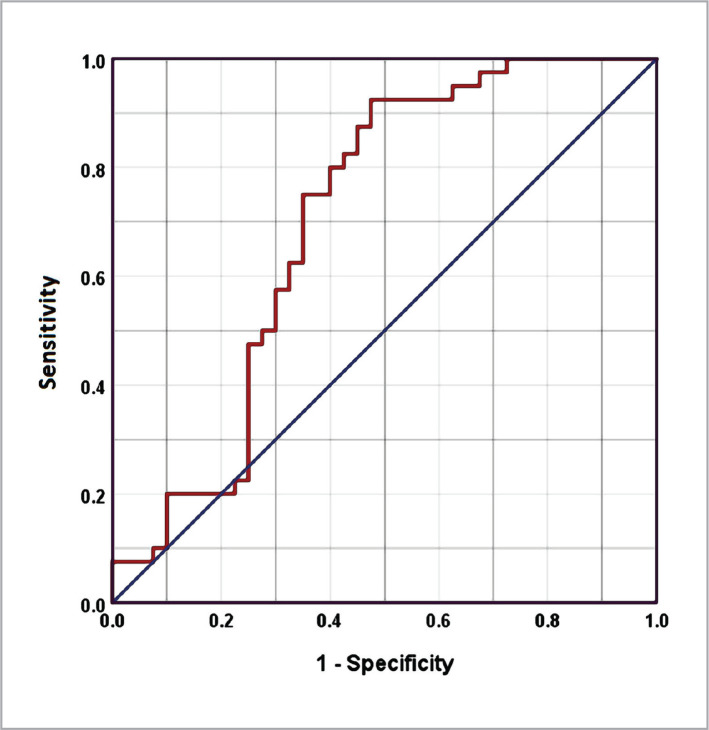
ROC curve for the combination of NfL and t-tau and neurofilament in the context of discrimination between patients with AD and controls.

Combining the two markers increased the AUC (0.703, 95%CI= 0.584-0.822). The sensitivity and specificity of the test at the above cut-off values were 75% and 60%, respectively (Figure 3).

### Correlation of ADAS-cog, NfL, and t-tau with age

We used Pearson's correlation to investigate the relationship between NfL, t-tau, and ADAS-cog with age and BMI in the patient group. T-tau protein showed a significant positive correlation with age (r=0.566, p<0.001), NfL (r=0.608, p<0.001), and ADAS-cog score (r=0.468, p=0.002). NfL correlated positively with age (r= 0.587, p<0.001) and ADAS-cog score (r=0.614, p<0.001). The ADAS-Cog score correlated positively with age (r=0.766, p<0.001) (Table 2).

**Table 2:** Pearson’s correlation of NfL, t-tau, and ADAS-Cog with age and BMI in patients with AD.

Variable	t-tau		NfL		ADAS-cog	
	Coefficient	P-value	Coefficient	P-value	Coefficient	P-value
**Age**	0.566	<0.001	0.587	<0.001	0.766	<0.001
**BMI**	-0.035	0.831	-0.136	0.403	-0.066	0.688
**NfL**	0.608	<0.001	-	-	-	-
**ADAS-Cog**	0.468	0.002	0.614	<0.001	-	-

ADAS-Cog – Alzheimer’s Disease Assessment Scale-Cognitive Subscale; NfL – Neurofilament light chain.

## DISCUSSION

The present study aimed to evaluate serum NfL and t-tau proteins in individuals diagnosed with AD dementia and determine their relationship with ADAS-cog. The study revealed a significantly higher ADAS-Cog score for patients with AD dementia compared to controls, suggesting that AD patients have moderate to severe cognitive impairment (CI) [14]. This result agrees with numerous studies worldwide [15,16].

Moreover, patients with AD dementia had considerably greater serum t-tau protein levels than controls, consistent with the results of numerous international studies [17-19]. Increased t-tau protein levels in cerebrospinal fluid (CSF) and plasma are shown to predict neuronal axon degeneration or injury, and higher t-tau protein levels of CSF were also reported in other situations, such as neuronal death following stroke [20]. Several studies have reported that plasma tau levels are a reflection of brain tau levels [19-21] and that individuals diagnosed with AD dementia exhibit markedly higher levels of plasma tau [22]. One study demonstrated a relation between plasma tau levels and the levels of t-tau or phosphorylated tau (p-tau), thereby emphasizing the usefulness of serum tau measurements for diagnosing AD dementia [23]. Furthermore, a recent study revealed that the levels of t-tau/Aβ1-42 in plasma are significantly predictive of brain tau deposition and are associated with long-term alterations in cerebral amyloid deposition, brain glucose metabolism, and changes in hippocampal volume [24,25].

According to the current study, there was a positive correlation between the ADAS-Cog score and serum t-tau level, consistent with the results of a previous study conducted in the United States [23]. A recent study indicated weakly significant relationships between serum t-tau levels and the Mini-mental State Examination (MMSE) and Global Deterioration Scale [26]. Other studies reported a strong correlation between elevated CSF tau protein in AD dementia and cognitive decline [27,28]. In our study, a plasma t-tau protein cutoff value of 20.61 pg/ml was found to predict AD dementia (AUC=0.69, 68% sensitivity, and 57% specificity). These findings were lower than those reported in an Indian study (AUC=0.907, 86.49% sensitivity, and 89.74% specificity) [18] and an American study (AUC=0.79; 76.7% sensitivity, and 81% specificity) [29], which stated that plasma t-tau protein is a predictor of AD dementia independent of CSF tau protein. Concerning NfL, its level was considerably elevated in patients with AD dementia. The results of numerous studies, including those from the USA [30] and China [31], reported a similar rise in patients with neurodegenerative disorders. Nevertheless, NfL is not disease-specific, with low predictability for AD dementia. Also, plasma NfL levels were higher in individuals with mild CI or AD dementia than in cognitively unimpaired individuals [32].

Conversely, a meta-analysis reported that the levels of NfL in both blood and CSF were unable to differentiate between AD dementia and other conditions that are similar to it, such as vascular dementia, dementia with Lewy bodies, Parkinson's disease dementia, idiopathic normal pressure hydrocephalus, and posterior cortical atrophy [33]. Elevated NfL levels may indicate the occurrence of neuroaxonal damage, which leads to their release into the extracellular space, and subsequently into the bloodstream and CSF. As a result, NfL can provide real-time information about neuroaxonal injury in the central nervous system (CNS) [34]. Moreover, the increasing and more varied levels of NfL in individuals >60 years imply a speeding up of neuronal injury, which may be driven by subclinical comorbid pathologies [35]. We found a positive relationship between serum NfL level and poor cognition estimated by the ADAS-Cog scale. Studies worldwide also noticed higher plasma NfL levels associated with poor cognition, increased risk of cognitive stage transition, brain atrophy, and brain hypo metabolism [32,36]. Additionally, it was observed that in familial cases of AD dementia, the levels of serum NfL were indicative of the rate of cognitive decline and cortical thinning [37]. Contrary to these findings, other studies found a strong negative correlation between serum NfL and cognitive measures in AD mutation carriers [38]. Considering the diagnostic value of NfL, we identified that a cut-off value of 11.21 pg/ml was a poor predictor of AD dementia (AUC=0.66, 68% sensitivity, and 52% specificity), which was lower than that found by a German study (cut-off value of 25.7 pg/ml, AUC=0.85, 84% sensitivity, and 78% specificity) [39]. Several factors could account for this variation, including differences in sample size and demographic characteristics of the patients.

## CONCLUSION

To summarize, the study demonstrated that while serum NfL and t-tau protein levels were higher in AD patients, they exhibited poor diagnostic performance in distinguishing AD from healthy controls. The study also found that age was significantly associated with the occurrence of AD biomarkers, including NfL, t-tau protein, and ADAS-Cog scores, suggesting that advanced age is a contributing factor to the increased prevalence of AD biomarkers. These results have important implications for the diagnosis and management of AD and underscore the need for more reliable and sensitive diagnostic markers for this disease.

## Data Availability

All data generated or analyzed during this study are included in this published article. The datasets used and/or analyzed during the current study are available from the corresponding author upon reasonable request.
